# Herbicide Safeners Decrease Sensitivity to Herbicides Inhibiting Acetolactate-Synthase and Likely Activate Non-Target-Site-Based Resistance Pathways in the Major Grass Weed *Lolium* sp. (Rye-Grass)

**DOI:** 10.3389/fpls.2017.01310

**Published:** 2017-08-08

**Authors:** Arnaud Duhoux, Fanny Pernin, Diane Desserre, Christophe Délye

**Affiliations:** l'Institut National de la Recherche Agronomique (INRA) Agroécologie, Dijon, France

**Keywords:** evolution, herbicide resistance, safener, plant secondary metabolism, transcriptional marker, weed management

## Abstract

Herbicides are currently pivotal to control weeds and sustain food security. Herbicides must efficiently kill weeds while being as harmless as possible for crops, even crops taxonomically close to weeds. To increase their selectivity toward crops, some herbicides are sprayed in association with safeners that are bioactive compounds exacerbating herbicide-degrading pathways reputedly specifically in crops. However, exacerbated herbicide metabolism is also a key mechanism underlying evolved non-target-site-based resistance to herbicides (NTSR) in weeds. This raised the issue of a possible role of safeners on NTSR evolution in weeds. We investigated a possible effect of the respective field rates of the two broadly used safeners cloquintocet-mexyl and mefenpyr-diethyl on the sensitivity of the troublesome global weed *Lolium* sp. (rye-grass) to the major herbicides inhibiting acetolactate-synthase (ALS) pyroxsulam and iodosulfuron + mesosulfuron, respectively. Three *Lolium* sp. populations were studied in three series of experiments. The first experiment series compared the frequencies of plants surviving application of each herbicide alone or in association with its safener. Safener co-application caused a net increase ranging from 5.0 to 46.5% in the frequency of plants surviving the field rate of their associated herbicide. In a second series of experiments, safener effect was assessed on individual plant sensitivity using vegetative propagation. A reduction in sensitivity to pyroxsulam and to iodosulfuron + mesosulfuron was observed for 44.4 and 11.1% of the plants in co-treatment with cloquintocet-mexyl and mefenpyr-diethyl, respectively. A third series of experiments investigated safener effect on the expression level of 19 *Lolium* sp. NTSR marker genes. Safeners showed an enhancing effect on the expression level of 10 genes. Overall, we demonstrated that cloquintocet-mexyl and mefenpyr-diethyl both reduced the sensitivity of *Lolium* sp. to their associated ALS-inhibiting herbicide and most likely exacerbated herbicide-degrading secondary metabolism pathways. This suggests that genetic variation for safener response is present in *Lolium* sp. Thus, a possible, uninvestigated way to NTSR evolution could be selection for increased responsiveness to safener action. Delivering safeners exclusively to the crop could mitigate the risk for NTSR evolution in weeds.

## Introduction

Agrestal weeds are a major threat to global crop yield (Oerke, 2006). Herbicide applications are currently the easiest and most efficient agricultural practice that can be implemented to restrain weed proliferation in cultivated fields. Herbicides are biologically active organic compounds disrupting vital plant physiological processes. Herbicides generally act by binding to one specific protein. To be agronomically and commercially successful, one herbicide must fulfill two antagonistic criteria, to wit, allowing efficient control of a broad range of weed species, including species taxonomically closely related to crops, while being as harmless as possible for crops. The necessity to compromise between powerful action on weeds and crop selectivity has been at the root of the success of herbicide antidotes for crops, or safeners (Rosinger, 2014). Like herbicides, safeners are bioactive organic compounds. When applied in association with herbicide(s), safeners have the intriguing capacity to decrease the sensitivity of one or several crop species enough to prevent herbicide damage without reducing in-field weed control efficiency. Accordingly, herbicides used in association with a safener represent ~30% of the value of global herbicide sales (Rosinger, 2014).

The agronomic importance of safeners has fostered considerable research on their physiological and biochemical mode of action, which has extensively been reviewed elsewhere (Davies and Caseley, 1999; Hatzios and Burgos, 2004; Riechers et al., 2010). Basically, the rapidity of herbicide metabolism in the plant is key for efficient herbicide action, i.e., one efficient herbicide kills the plant before the plant has time to metabolize it. Safeners decrease herbicide sensitivity in safener-responsive plant species by accelerating the metabolism of herbicides into less active or inactive compounds. Safeners cause a coordinated increase in the intracellular contents in proteins or enzymes involved in the successive phases of multistep xenobiotic metabolism by triggering an up-regulation of the transcription of the corresponding genes. While the signaling pathways involved in the regulation of plant response to safeners remain unclear, protein and enzyme families induced in response to safener action have been identified. These families are involved in the four successive phases of herbicide metabolism (Kreuz et al., 1996; Hatzios and Burgos, 2004; Zhang et al., 2007). They include cleaving or oxidizing enzymes (e.g., cytochromes P450, hydrolases or esterases, phase I), conjugating enzymes (e.g., glycosyl-transferases or glutathione-*S*-transferases, phase II), conjugate metabolite transporters (e.g., ABC transporters, phase III), and enzymes involved in conjugated metabolite degradation (e.g., carboxypeptidases, phase IV).

The metabolic pathways involved in accelerated herbicide degradation that are induced by safeners in crop plants are strikingly similar to those involved in non-target-site-based resistance to herbicides (NTSR) in weeds (Yuan et al., 2007; Délye, 2013). NTSR can evolve in weed populations in response to the selective pressure exerted by herbicide applications in the field (Délye et al., 2013). Among the different mechanisms that can underlie NTSR, enhanced metabolism causing accelerated herbicide degradation is a major cause for NTSR (Yuan et al., 2007; Délye, 2013). However, as safeners have reputedly little or no action on weeds, the vast majority of studies aiming at elucidating their mode of action have targeted crop or model plant species (Davies and Caseley, 1999; Hatzios and Burgos, 2004; Riechers et al., 2010). Only a few studies investigated the possible effects of safeners on non-crop or weed species (Cummins et al., 1999; Brazier et al., 2002; Reade et al., 2004; Del Buono et al., 2007; Del Buono and Ioli, 2011). The weed species most intensively studied regarding safener action on herbicide sensitivity is *Alopecurus myosuroides* (black-grass). One previous study had shown that co-application of the acetolactate-synthase (ALS, or acetohydroxyacid-synthase, EC 2.2.1.6) inhibitor pyroxsulam and its safener cloquintocet-mexyl caused a non-significant trends toward accelerated metabolism of pyroxsulam in herbicide-sensitive *A. myosuroides* plants (deBoer et al., 2011). Another study reported a decrease in sensitivity to the ALS inhibitors iodosulfuron and mesosulfuron in *A. myosuroides* plants from field populations, regardless of the presence of resistance (Rosenhauer et al., 2016). More detailed biochemical studies clearly demonstrated that safeners decreased sensitivity to one herbicide inhibiting acetyl-CoA carboxylase (ACCase, EC 6.4.1.2) in herbicide-sensitive *A. myosuroides* plants and could also exacerbate NTSR in herbicide-resistant plants (Cummins et al., 2009). Safener effects were found functionally similar to NTSR, although NTSR involved fewer metabolic changes than response to safeners and was accordingly postulated to involve a narrower set of metabolic pathways (Cummins et al., 2009, 2013). Overall, all these studies suggested that safeners had an activating effect on metabolic pathways involved in NTSR to ACCase inhibitors in the grass weed *A. myosuroides*, raising the issue of a possible facilitating effect of safeners on the evolution of NTSR in grass weeds.

Rye-grasses (*Lolium* spp.) are among the most troublesome weeds globally (Heap, 2017). Control of *Lolium* spp. in winter cereals where they are particularly troublesome chiefly relies on applications of herbicides inhibiting ALS. ALS inhibitors active on grass weeds are are applied together with safeners because they are not selective of cereal crops (Hacker et al., 2000; Brink et al., 2002; Becker et al., 2008). Recurrent application of formulated mixtures of ALS inhibitors and safeners have selected for NTSR mechanisms in numerous *Lolium* sp. populations worldwide (Heap, 2017). Yet, possible links between safener and NTSR to ALS inhibitors had not been investigated to date in *Lolium* sp. Actually, except in two studies addressing *A. myosuroides* (deBoer et al., 2011; Rosenhauer et al., 2016), safener effects on weed sensitivity to ALS inhibitors had not been investigated previously. Our aim was to investigate a possible effect of safeners on *Lolium* sp. sensitivity to ALS inhibitors and on NTSR to these herbicides. For this purpose, we assessed the effect of two major safeners on *Lolium* sp. phenotypic sensitivity to ALS inhibitors. We also measured the effect of safener application on the expression level of recently identified genes that are associated to NTSR to ALS inhibitors in *Lolium* sp. (Duhoux et al., 2015, 2017).

## Materials and methods

### Plant material selection

Resistance or sensitivity to herbicides are observed at the individual plant level. In the case of *Lolium* sp., resistance to ALS inhibitors can be mediated by NTSR and/or by mutations at the gene encoding ALS (target-site-based resistance, see Délye et al., 2013). To investigate a possible effect of safeners on *Lolium* sp. sensitivity to ALS inhibitors, we sought populations comprising contrasted frequencies of plants resistant to the rates of ALS-inhibiting herbicides applied in the field because of NTSR. One preliminary experiment was therefore conducted to identify *Lolium* sp. populations suitable for our purpose. The herbicides considered were the two major commercial formulations of ALS inhibitors used against *Lolium* sp. The first one was Archipel, a water-dispersible granule formulation containing 3.0% (weight/weight) of each of the two sulfonylurea herbicides iodosulfuron and mesosulfuron and 9.0% (weight/weight) of the safener mefenpyr-diethyl (Bayer CropScience, Lyon, France). The second herbicide studied was Abak, a water-dispersible granule formulation containing 7.5% (weight/weight) of the triazolopyrimidine herbicide pyroxsulam and 7.5% (weight/weight) of the safener cloquintocet-mexyl (Dow AgroSciences, Valbonne, France). In all experiments described thereafter, pyroxsulam, iodosulfuron + mesosulfuron and their respective safeners cloquintocet-mexyl and mefenpyr-diethyl were applied at their French recommended field rates and in conditions mimicking application in the field to assess the possible effect of safeners on *Lolium* sp. sensitivity to herbicides in conditions as close as possible to those prevailing in agricultural fields.

For each of two dozen of *Lolium* sp. populations, two batches of 50 seedlings each and one batch of 25 seedlings were grown in a glasshouse at 22°C/18°C day/night with 14-h photoperiod in containers (17 × 12.5 × 5.5 cm in dimensions; 25 seedlings per container) filled with a mixture of soil (1/3), sand (1/3), and compost (1/3) until the 3-4-leaf stage at which ALS-inhibiting herbicide application is recommended. For every population, one batch of 50 plants was sprayed with the French recommended field rate of Archipel (7.5 g iodosulfuron + 7.5 g mesosulfuron + 22.5 g mefenpyr-diethyl ha^−1^) and the second one with the French recommended field rate of Abak (18.75 g pyroxsulam + 18.75 g cloquintocet-mexyl ha^−1^). An adjuvant enhancing herbicide penetration into leaf tissues (Actirob B; Bayer CropScience, 1 L ha^−1^) was added in the spraying mix, as recommended by the manufacturers. The batch of 25 plants was sprayed with water (untreated control). The herbicide application procedure was as described (Petit et al., 2012).

Plant phenotypes were visually rated 4 weeks after application. Plants killed were rated sensitive, while surviving plants were rated resistant. To exclude populations with plants containing mutant, herbicide-resistant ALS alleles from subsequent experiments, all plants rated “resistant” were subjected to ALS genotyping as described (Délye et al., 2009). This procedure allowed us to select three *Lolium* sp. populations with contrasted frequencies of plants resistant to Archipel and/or Abak because of NTSR (Table 1).

**Table 1:** Field rye-grass populations studied.

**Population code**	**Year collection**	**Origin (French département)**	**% Plants resistant to the commercial herbicide:**	
			**Abak (pyroxsulam + cloquintocet-mexyl)**	**Archipel (iodosulfuron + mesosulfuron + mefenpyr-diethyl)**
RG07-044	2007	Tarn	24.0	5.0
RG07-046	2007	Aisne	96.5	60.0
RG12-069	2012	Rhône	64.5	35.0

### Experiment series 1: effect of safeners on the observed frequencies of resistant plants in *Lolium* sp. populations

To assess the effect of cloquintocet-mexyl on *Lolium* sp. sensitivity to pyroxsulam, 75 seedlings from each of the three populations studied were sprayed with pyroxsulam + cloquintocet-mexyl at 18.75 g ha^−1^ each (French recommended field rate) and 75 additional seedlings per population were sprayed with pyroxsulam at 18.75 g ha^−1^. The water-dispersible granule formulation of the commercial herbicide Abak was used for pyroxsulam + cloquintocet-mexyl and for pyroxsulam alone, so that no bias due to the herbicide formulation was introduced in the experiment. The adjuvant Actirob (1 L ha^−1^) was added to the two respective spraying mixes. Twenty-five additional seedlings per population were sprayed with water (untreated control).

A similar experimental design was used to assess the effect of mefenpyr-diethyl on *Lolium* sp. sensitivity to iodosulfuron + mesosulfuron. As no iodosulfuron + mesosulfuron formulated without mefenpyr-diethyl could be obtained, iodosulfuron, mesosulfuron, and mefenpyr-diethyl were purchased at the analytical standard grade (Sigma-Aldrich, USA). A 2% (volume/volume) dilution of ethoxylated castor oil (polyoxyl 35 castor oil, Sigma-Aldrich) was used as a spraying formulation. The experiment consisted into three modalities each including 75 seedlings per population. The first modality was sprayed with iodosulfuron + mesosulfuron + mefenpyr-diethyl at 7.5 g + 7.5 g + 22.5 g ha^−1^, respectively, in castor oil. The second modality was sprayed with iodosulfuron + mesosulfuron at 7.5 g ha^−1^ each in castor oil. The third modality was sprayed with the commercial herbicide Archipel at the French recommended field rate (iodosulfuron + mesosulfuron + mefenpyr-diethyl at 7.5 g + 7.5 g + 22.5 g ha^−1^, respectively) as a check for the relevance of the castor oil formulation. The adjuvant Actirob (1 L ha^−1^) was added to the three respective spraying mixes. Twenty-five additional seedlings per population were sprayed with water (untreated control).

Both experiments were replicated. In all experiments, herbicide or water application and visual phenotype rating 4 weeks after application were as described in the preceding section.

### Production of plant material for experiment series 2 (phenotypic effects of safeners on individual *Lolium* sp. plants) and 3 (transcriptional effect of safeners)

**Figure 1:**
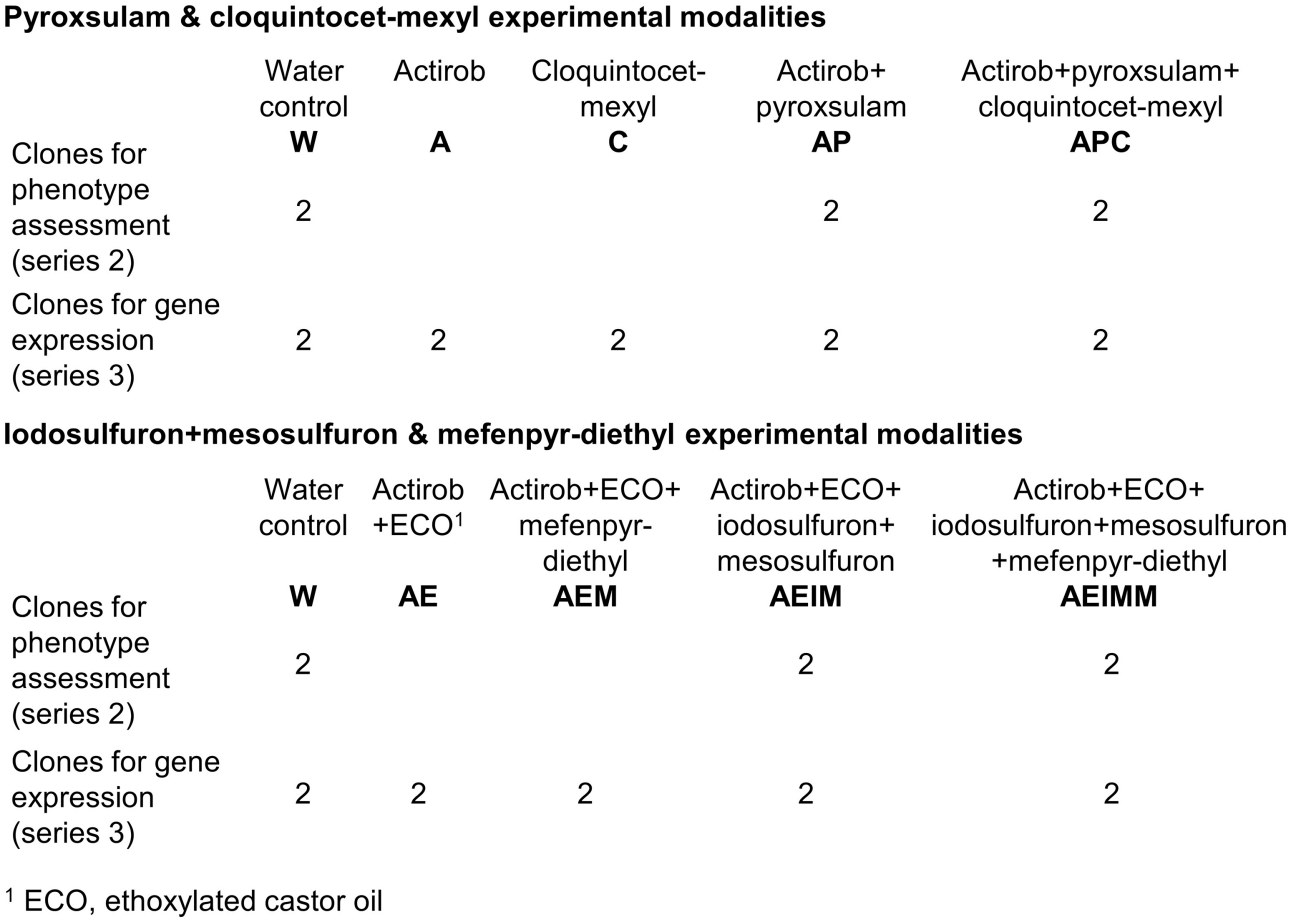
Distribution of the 16 clones per rye-grass plant studied among the experimental modalities of the two series of spraying experiments used to produce plant material to investigate safener effect on individual plant phenotype (experiment series 2) and on NTSR marker gene expression (experiment series 3).

For each of the three populations studied, one batch of 12 plants was used to study the effect of cloquintocet-mexyl on individual plant sensitivity to pyroxsulam (experiment series 2) and on the expression of *Lolium* sp. NTSR marker genes (experiment series 3). Another batch of 12 plants was used to study the effect of mefenpyr-diethyl on individual plant sensitivity to iodosulfuron + mesosulfuron (experiment series 2) and on the expression of *Lolium* sp. NTSR marker genes (experiment series 3). Plants were grown in individual 2 L-pots in a glasshouse at 22°C/18°C day/night with 14-h photoperiod. At the sixteen-tiller stage, they were subjected to vegetative propagation: all individual tillers were separated and transplanted into individual pots. For each plant, this yielded 16 clones (genetic replicates) at the 3-4-leaf growth stage. The distribution of the 16 clones per plant over the two series of experiments is summarized in Figure 1.

The batches of cloned plants intended for cloquintocet-mexyl effect investigation were sprayed according to five modalities with every compound applied at the French field rate (Figure 1). Modalities included water-sprayed (W), Actirob applied at 1 L ha^−1^ (A), cloquintocet-mexyl applied at 18.75 g ha^−1^ (C), pyroxsulam applied at 18.75 g ha^−1^ with Actirob at 1 L ha^−1^ (AP) and pyroxsulam and cloquintocet-mexyl applied at 18.75 g ha^−1^ with Actirob at 1 L ha^−1^ (APC). The water-dispersible granule formulation of the commercial herbicide Abak was used for modalities APC, AP, and C so that no bias due to the herbicide formulation was introduced in the experiment. Four clones per plant were included in modalities UT, AP, and APC, and two clones per plant in the other two modalities. Two clones per plant and per modality were intended for RNA extraction (clones for experiment series 3, Figure 1). The remaining two clones in each of modalities W, AP, and APC were used to detect shifts in herbicide sensitivity of individual plants caused by the presence of the safener by comparing the phenotypes of clones sprayed with the pyroxsulam alone (AP) or in association with cloquintocet-mexyl (APC) (clones for experiment series 2, Figure 1).

The batches of cloned plants intended for mefenpyr-diethyl effect investigation were also sprayed according to five modalities with every compound applied at the French field rate (Figure 1). Modalities included water-sprayed (W), ethoxylated castor oil at 2% volume/volume + Actirob at 1 L ha^−1^ (AE), ethoxylated castor oil at 2% volume/volume + mefenpyr-diethyl at 22.5 g ha^−1^ + Actirob at 1 L ha^−1^ (AEM), ethoxylated castor oil at 2% volume/volume + iodosulfuron + mesosulfuron at 7.5 g ha^−1^ each + Actirob at 1 L ha^−1^ (AEIM) and ethoxylated castor oil at 2% volume/volume + iodosulfuron + mesosulfuron at 7.5 g ha^−1^ each + mefenpyr-diethyl at 22.5 g ha^−1^ + Actirob at 1 L ha^−1^ (AEIMM). As for the preceding plant batches, four clones per plant were included in modalities AE, AEIM, and AEIMM and two clones per plant in the other two modalities. Two clones per plant and per modality were intended for RNA extraction (clones for experiment series 3, Figure 1). The remaining two clones in each of modalities AE, AEIM, and AEIMM were used to detect shifts in herbicide sensitivity of individual plants caused by the presence of the safener by comparing the phenotypes of clones sprayed with iodosulfuron + mesosulfuron alone (AEIM) or in association with mefenpyr-diethyl (AEIMM) (clones for experiment series 2, Figure 1).

Spraying was as described in the preceding section for both batches.

### Experiment series 2: phenotypic effects of safeners on individual *Lolium* sp. plants

The phenotypes of the clones of each plant in each modality of the pyroxsulam and cloquintocet-mexyl experiment and of the iodosulfuron + mesosulfuron and mefenpyr-diethyl experiment used for phenotype assessment (Figure 1) were visually rated 4 weeks after spraying. Clones killed were rated sensitive (S). Clones markedly affected but surviving and growing were rated moderately resistant (M). Clones unaffected or moderately affected were rated resistant (R). Plants were subsequently assigned to one of four phenotype classes according to the phenotypic rating of the clones sprayed with the herbicide alone or associated to its safener. Class A included plants whose clones were rated S or M in the presence and in the absence of safener. Class B included plants whose clones showed a moderate decrease in sensitivity in the presence of the safener (from S to M or from M to R). Class C included plants whose clones showed a substantial decrease in sensitivity in the presence of the safener (from S to R). Class D included plants whose clones were rated R in the presence and in the absence of safener.

### Experiment series 3: transcriptional effects of safeners

ALS inhibitors essentially act in the meristems (Zhou et al., 2007) that are at the leaf basis in grasses. Twenty-four hours after spraying, the basis of the above-ground part of the clones of each plant in each modality of the pyroxsulam and cloquintocet-mexyl experiment and of the iodosulfuron + mesosulfuron and mefenpyr-diethyl experiment intended for studying safener transcriptional effects (Figure 1) was cut, placed in one 2-mL tube, immediately frozen in liquid nitrogen and stored at −80°C until RNA extraction. The 24-h time point was chosen because previous results suggested that the expression level of NTSR pathways within the 48 h following ALS inhibitor application were crucial to determine plant survival (Duhoux et al., 2017).

The pyroxsulam and cloquintocet-mexyl experiment and the iodosulfuron + mesosulfuron and mefenpyr-diethyl experiment each yielded 360 samples for RNA extraction (12 plants × 3 population × 5 experimental modalities × two clones per plant, Figure 1). Total RNA was extracted from each sample and RT-qPCR was used to measure the expression level of 19 NTSR transcriptional markers in all samples as described (Duhoux et al., 2017). NTSR markers are genes with a constitutive significantly higher expression level in plants with NTSR compared to sensitive plants. NTSR markers enabled identification of plants with NTSR on the basis of their expression data (Duhoux et al., 2017). Expression level of each NTSR marker was measured separately in each of the two clones from each plant in each experimental modality. The average expression value obtained for each pair of clones was used in subsequent analyses.

### Statistical analyses

Statistical analyses were performed using R version 3.1.2 (http://www.r-project.org). To investigate the effects of each herbicide assayed and its associated safener on NTSR marker expression levels, an ANOVA was performed using a model that included the effects of the experimental modality, of the population and of their interaction as fixed factors. Tukey's test was implemented for multiple comparisons of averaged expression level values.

## Results

### Effect of safeners on the frequency of resistant plants in *Lolium* sp. populations (experiment series 1)

**Figure 2:**
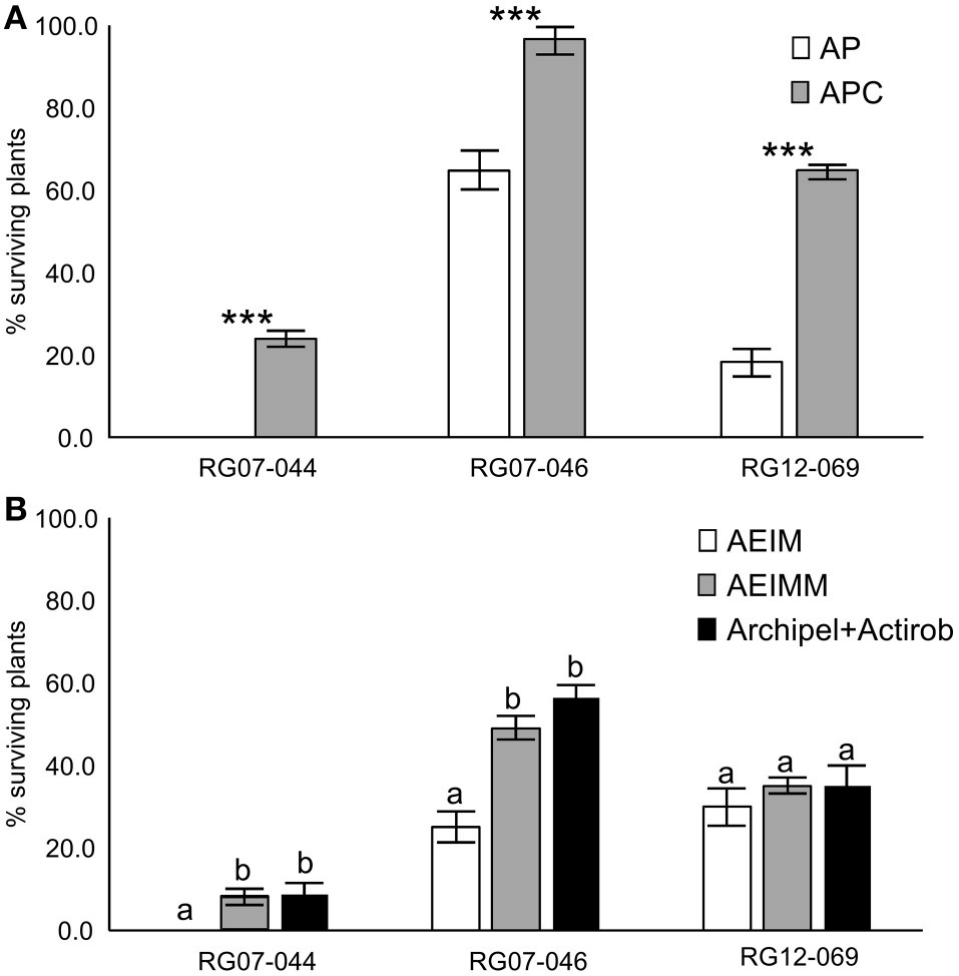
Frequencies of resistant plants in the three rye-grass populations studied. **(A)** Frequencies of resistant plants in the three rye-grass populations studied observed after the application of pyroxsulam + Actirob (AP, white bars) or pyroxsulam + cloquintocet-mexyl + Actirob (APC, gray bars). ^***^Indicate significant differences in the number of resistant plants between experimental modalities within each population according to Fisher's exact test (*p* < 0.001). **(B)** Frequencies of resistant plants in the three rye-grass populations studied observed after the application of iodosulfuron + mesosulfuron in ethoxylated castor oil + Actirob (AEIM, white bars), iodosulfuron + mesosulfuron + mefenpyr-diethyl in ethoxylated castor oil + Actirob (AEIMM, gray bars) and the commercial herbicide Archipel + Actirob (Archipel + Actirob, black bars). Different letters among experimental modalities within one population indicate significant differences in the number of resistant plants according to Fisher's exact test (*p* < 0.05).

The joint application of each safener studied and of its associated herbicide caused a shift in the frequencies of surviving plants in the three populations studied compared to the application of the herbicide alone (Figure 2). Joint application of cloquintocet-mexyl and pyroxsulam resulted into a significantly higher frequency of surviving plants in all three *Lolium* sp. populations studied compared to the application of pyroxsulam alone (Figure 2A). The net increase in the frequency of surviving plants ranged from 23.8 to 46.5%. The most obvious shift was observed in population RG07-044 where the frequency of surviving plants increased from 0.0% when pyroxsulam was applied alone to 23.8% when cloquintocet-mexyl was associated to pyroxsulam.

In the iodosulfuron + mesosulfuron experiment, the frequencies of surviving plants identified in each population using the castor oil formulation including iodosulfuron + mesosulfuron and mefenpyr-diethyl were not significantly different from those identified using the commercial herbicide Archipel (Figure 2B). Joint application of mefenpyr-diethyl and iodosulfuron + mesosulfuron in castor oil resulted into a significantly higher frequency of surviving plants in two of the three *Lolium* sp. populations studied compared to the application of iodosulfuron + mesosulfuron alone in castor oil. The net increase in the frequency of surviving plants ranged from 5.0 to 24.0%. In population RG07-044, the frequency of surviving plants increased from 0.0% when iodosulfuron + mesosulfuron were applied alone to 10.0% when mefenpyr-diethyl was associated to iodosulfuron + mesosulfuron.

### Effect of safeners on the herbicide sensitivity of individual *Lolium* sp. plants (experiment series 2)

**Figure 3:**
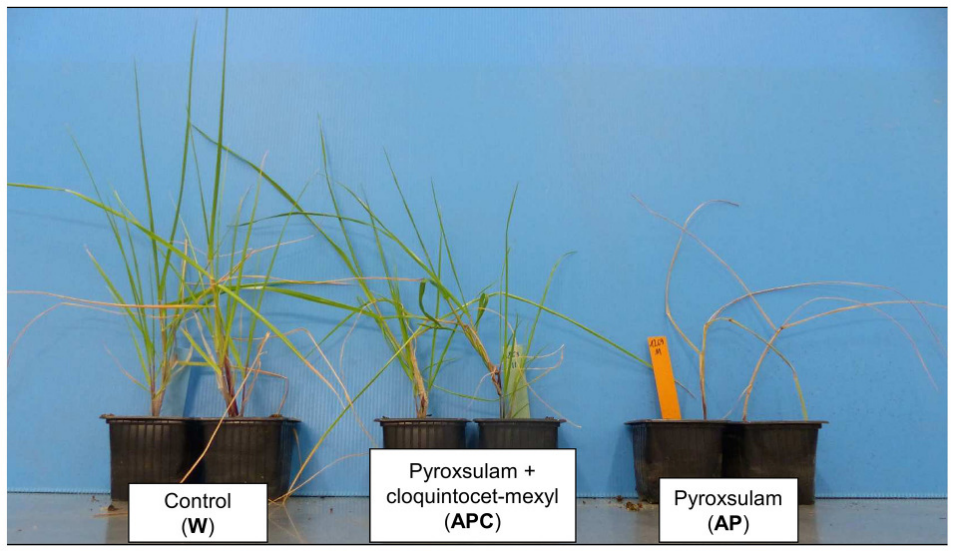
Phenotypes of the clones of one rye-grass plant from population RG12-069 in the three experimental modalities used to assess the effect of the safener cloquintocet-mexyl on the sensitivity of individual rye-grass plants to pyroxsulam. The clones from this plant were rated sensitive (S) to pyroxsulam (AP) and resistant (R) to pyroxsulam associated to cloquintocet-mexyl (APC). W, water-sprayed control.

The effect of each safener studied on the sensitivity of individual plants to the associated herbicide was assessed using 12 plants per safener and per population studied. Every plant was assigned to one of four phenotype classes according to the respective phenotypic ratings of the clones sprayed with the herbicide alone or associated to its safener. Both clones from every plant showed identical phenotypes within one given experimental modality (illustrated in Figure 3). Plants whose clones were rated sensitive or moderately resistant to the herbicide in the presence and in the absence of its safener were assigned to class A. Plants whose clones showed a moderate decrease in sensitivity to the herbicide in the presence of its safener (from sensitive to moderately resistant or from moderately resistant to resistant) were assigned to class B. Plants whose clones showed a substantial decrease in sensitivity to the herbicide in the presence of its safener (from sensitive to resistant, illustrated in Figure 3) were assigned to class C. Plants whose clones were rated resistant to the herbicides in the presence and in the absence its safener were assigned to class D. We did not observe a visible increase in herbicide sensitivity in the presence of the safener for any plant or herbicide studied.

In the pyroxsulam and cloquintocet-mexyl experiment, a total of 16 plants (44.4% of the plants assayed) showed a reduction in sensitivity to pyroxsulam in the presence of cloquintocet-mexyl, of which three shifted from sensitive to fully resistant (Table 2; phenotype change illustrated in Figure 3). Nine of these plants, including two of the three plants that shifted from sensitive to fully resistant, originated from population RG12-069 that showed an intermediate frequency of plants resistant to the commercial formulation of pyroxsulam + cloquintocet-mexyl (Table 1).

**Table 2:** Assignation to phenotype classes of the rye-grass plants used to assess the effect of safeners on the sensitivity of individual plants to the associated herbicide.

**Population**	**Phenotype class**^**a**^			
	**A**	**B**	**C**	**D**
**PYROXSULAM AND CLOQUINTOCET-MEXYL EXPERIMENT**				
RG07-044	8	3	1	0
RG12-069	1	7	2	2
RG07-046	1	3	0	8
Total	10	13	3	10
**IODOSULFURON**+**MESOSULFURON AND MEFENPYR-DIETHYL EXPERIMENT**				
RG07-044	12	0	0	0
RG12-069	7	1	0	4
RG07-046	6	2	1	3
Total	25	3	1	7

a *Twelve plants were studied per population and per herbicide. Phenotype classes: A, plants whose clones were rated sensitive or moderately resistant in the presence and in the absence of safener, B, plants whose clones showed a moderate decrease in sensitivity in the presence of the safener (from S to M or from M to R); C, plants whose clones showed a substantial decrease in sensitivity in the presence of the safener (from S to R); D, plants whose clones were rated M or R in the presence and in the absence of safener*.

In the iodosulfuron + mesosulfuron and mefenpyr-diethyl experiment, a total of four plants (11.1% of the plants assayed) showed a reduction in sensitivity to iodosulfuron + mesosulfuron in the presence of mefenpyr-diethyl, of which one shifted from sensitive to fully resistant (Table 2). Three of these plants, including the one that shifted from sensitive to fully resistant, originated from population RG07-046 that showed the highest frequency of plants resistant to the commercial formulation of iodosulfuron + mesosulfuron + mefenpyr-diethyl (Table 1).

### Effect of safeners on the expression level of NTSR marker genes (experiment series 3)

The expression level of 19 NTSR marker genes (Duhoux et al., 2017) was measured 24 h after application of the experimental modalities in the pyroxsulam and cloquintocet-mexyl experiment and in the iodosulfuron + mesosulfuron and mefenpyr-diethyl experiment.

ANOVA identified a significant effect of the experimental modality on the expression level of 15 of the 19 NTSR markers in both the pyroxsulam and cloquintocet-mexyl experiment and the iodosulfuron + mesosulfuron and mefenpyr-diethyl experiment (Table 3). There was also a significant effect of the experimental modality on the expression level of two additional markers in the iodosulfuron + mesosulfuron and mefenpyr-diethyl experiment only. No effect of the experimental modality on the expression level of the two remaining markers was detected. A significant effect of the *Lolium* sp. population was detected on the expression level of four markers in both experiments, of two additional markers in the pyroxsulam and cloquintocet-mexyl experiment only and of six additional markers in the iodosulfuron + mesosulfuron and mefenpyr-diethyl experiment only. However, the interaction between experimental modality and population was never significant (Table 3).

**Table 3:** ANOVA of the effects of the experimental modality, of the population and of their interaction on the expression levels of 19 NTSR markers.

**Marker code**	**Pyroxsulam** + **cloquintocet-mexyl experiment**			**Iodosulfuron** + **mesosulfuron** + **mefenpyr-diethyl experiment**		
	**Factor**^**a**^			**Factor**^**a**^		
	**Experimental modality**	**Population**	**Experimental modality × population**	**Experimental modality**	**Population**	**Experimental modality × population**
*ABC-A*	1.44 × 10^−13^^***^	NS	NS	2.00 × 10^−14^^***^	8.96 × 10^−6^^***^	NS
*ABC-B*	1.80 × 10^−14^^***^	NS	NS	1.90 × 10^−14^^***^	NS	NS
*ALDOL-B*	3.37 × 10^−10^^***^	NS	NS	5.13 × 10^−14^^***^	NS	NS
*CYP72A*	2.00 × 10^−14^^***^	NS	NS	1.80 × 10^−14^^***^	NS	NS
*CYP72A2*	1.90 × 10^−14^^***^	NS	NS	1.70 × 10^−14^^***^	5.99 × 10^−4^^***^	NS
*CYP81B1*	5.59 × 10^−9^^***^	NS	NS	4.73 × 10^−10^^***^	1.13 × 10^−2^^*^	NS
*CYP81B2*	5.86 × 10^−10^^***^	NS	NS	2.96 × 10^−11^^***^	9.87 × 10^−4^^***^	NS
*ESTERA*	NS	NS	NS	4.38 × 10^−3^^**^	NS	NS
*GST-phi-A*	6.29 × 10^−5^^***^	3.58 × 10^−8^^***^	NS	1.60 × 10^−14^^***^	6.50 × 10^−3^^**^	NS
*GST-tau-I*	NS	1.08 × 10^−3^^**^	NS	2.62 × 10^−6^^***^	6.05 × 10^−4^^***^	NS
*GST-tau-J*	1.05 × 10^−7^^***^	1.07 × 10^−3^^**^	NS	8.90 × 10^−13^^***^	2.91 × 10^−10^^***^	NS
*GST-tau-K*	NS	NS	NS	NS	NS	NS
*GT-A*	NS	1.90 × 10^−3^^**^	NS	NS	NS	NS
*GT-C*	1.81 × 10^−14^^***^	1.03 × 10^−6^^***^	NS	1.50 × 10^−14^^***^	4.17 × 10^−2^^*^	NS
*GT-D*	1.10 × 10^−12^^***^	NS	NS	1.40 × 10^−14^^***^	6.39 × 10^−3^^**^	NS
*HYDROL-A*	3.46 × 10^−12^^***^	NS	NS	2.68 × 10^−8^^***^	NS	NS
*HYDROL-B*	1.80 × 10^−10^^***^	NS	NS	1.30 × 10^−14^^***^	NS	NS
*PEPTIDA*	1.70 × 10^−14^^***^	NS	NS	1.20 × 10^−14^^***^	6.09 × 10^−5^^***^	NS
*TRANSF*	4.87 × 10^−12^^***^	1.58 × 10^−2^^*^	NS	1.10 × 10^−14^^***^	NS	NS

a Significance of the factor effect: NS, not significant (F ≥ 0.05);

* F < 0.05;

** F < 0.01;

*** *F < 0.001 after sequential Bonferroni correction*.

**Figure 4:**
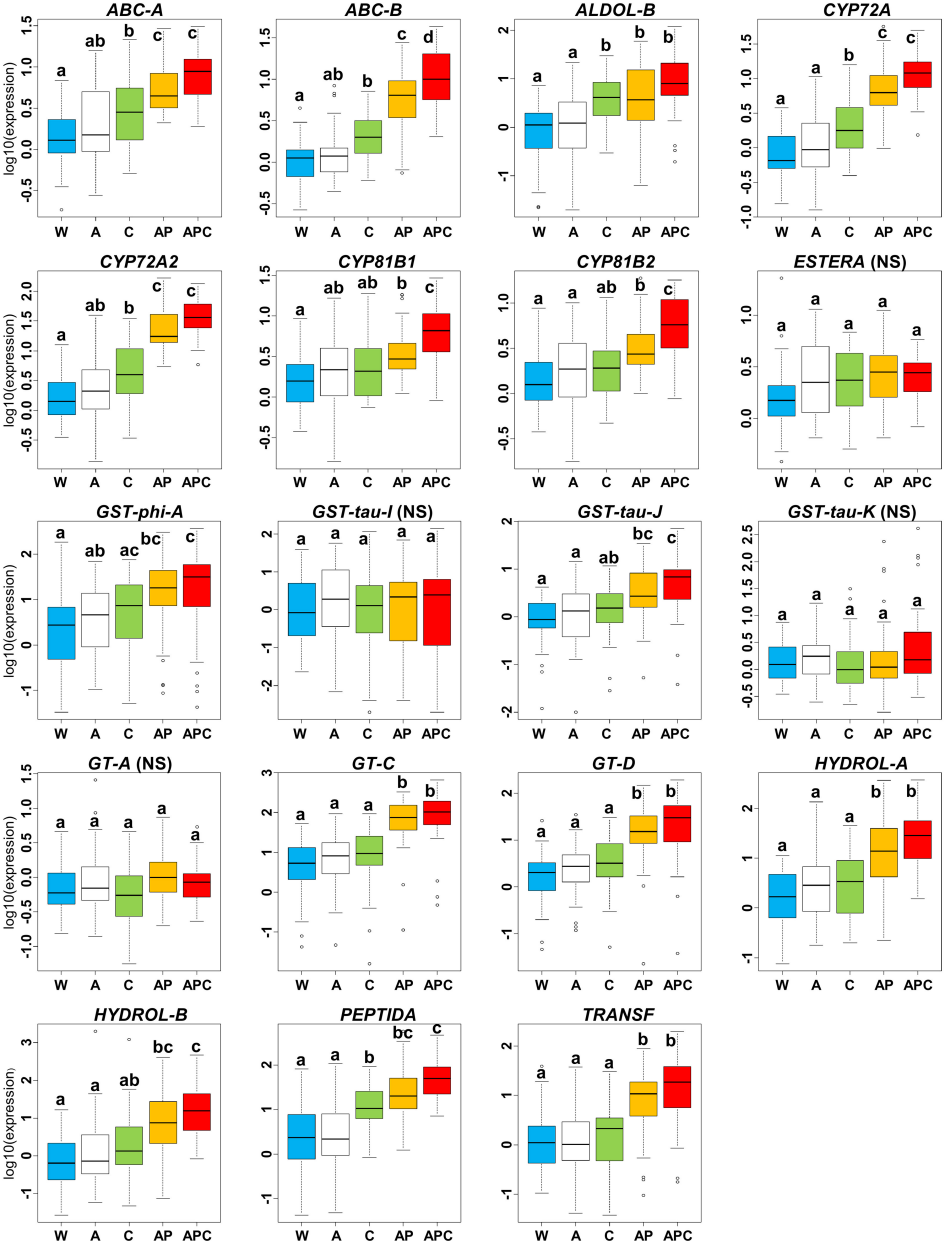
Variation of relative expression levels of 19 NTSR marker genes among experimental modalities in the pyroxsulam and cloquintocet-mexyl experiment. Expression levels 24 h after treatment are averaged over the 36 rye-grass plants studied. Experimental modalities: W, water; A, Actirob (adjuvant recommended for herbicide application); C, cloquintocet-mexyl; AP, pyroxsulam + Actirob; APC, pyroxsulam + cloquintocet-mexyl + Actirob. For each marker, different letters indicate significantly different expression levels (Tukey's test, *p* < 0.05). NS, marker for which no significant effect of the experimental modality was detected in ANOVA (Table 3).

In the pyroxsulam and cloquintocet-mexyl experiment, there was no significant effect of the herbicide adjuvant Actirob on the average expression level of NTSR markers computed over the 36 *Lolium* sp. plants studied (comparison of modalities A vs. W, Figure 4). Considering the 15 markers for which a significant effect of the experimental modality had been detected on the expression level in this experiment (Table 3), the average expression level of 13 markers was significantly higher in the presence of pyroxsulam (comparison of modalities AP vs. A, Figure 4). For these markers, the average increase in expression level ranged from 3.0-fold (*CYP81B1*) to 31.3-fold (*PEPTIDA*). A possible effect of the safener cloquintocet-mexyl on the expression level of the 15 markers was sought in two pairs of experimental modalities: cloquintocet-mexyl alone (without pyroxsulam and Actirob) vs. water (comparison of modalities C vs. W, Figure 4) and Actirob + pyroxsulam vs. Actirob + pyroxsulam + cloquintocet-mexyl (comparison of modalities APC vs. AP, Figure 4). A significantly higher expression in the presence of cloquintocet-mexyl was observed in both modality comparisons for *ABC-B* (2.7-fold and 2.9-fold increase in expression level, respectively). A significantly higher expression level in the presence of cloquintocet-mexyl was observed only in the APC-AP modality comparison for two markers (*CYP81B1* and *CYP81B2*; 2.9-fold and 2.8-fold increase in expression level, respectively). A significantly higher expression level in the presence of cloquintocet-mexyl was observed only in the comparison of modalities C vs. W for five markers (*ABC-A, ALDOL-B, CYP72A, CYP72A2*, and *PEPTIDA*). For these markers, the average increase in expression level ranged from 2.9-fold (*ABC-A*) to 30.5-fold (*ALDOL-B*).

**Figure 5:**
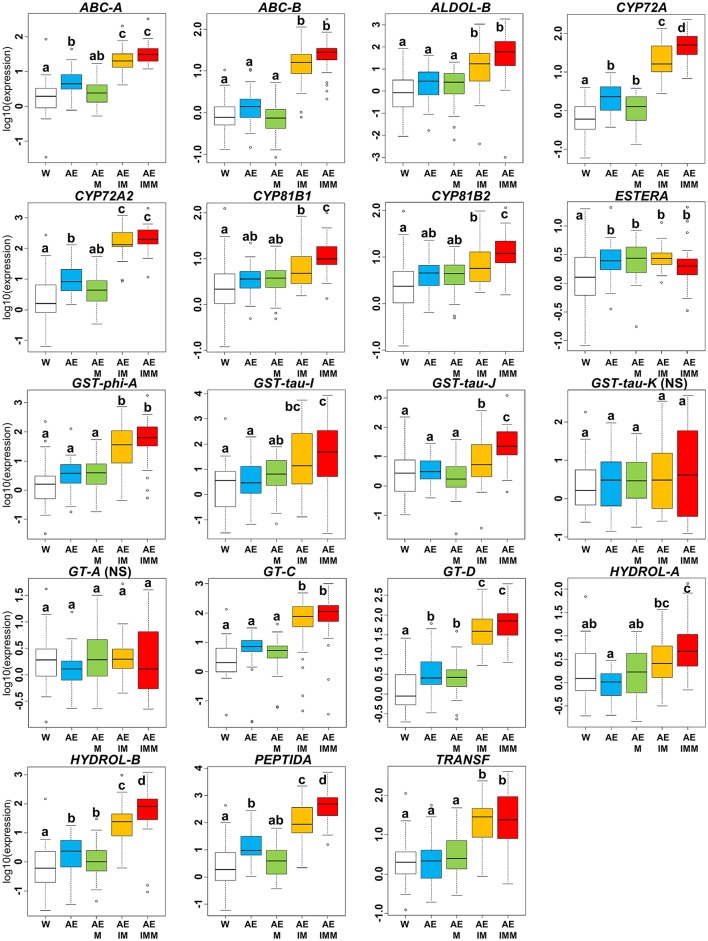
Variation of relative expression levels of 19 NTSR marker genes among experimental modalities in the iodosulfuron + mesosulfuron and mefenpyr-diethyl experiment. Expression levels 24 h after treatment are averaged over the 36 rye-grass plants studied. Experimental modalities: W, water; AE, Actirob (adjuvant recommended for herbicide application) + ethoxylated castor oil; AEM, Actirob + ethoxylated castor oil + mefenpyr-diethyl; AEIM; Actirob + ethoxylated castor oil + iodosulfuron + mesosulfuron; AEIMM, Actirob + ethoxylated castor oil + iodosulfuron + mesosulfuron + mefenpyr-diethyl. For each marker, different letters indicate significantly different expression levels (Tukey's test, *p* < 0.05). NS, marker for which no significant effect of the experimental modality was detected in ANOVA (Table 3).

In the iodosulfuron + mesosulfuron and mefenpyr-diethyl experiment, there was a significant effect of the mixture of ethoxylated castor oil and of the adjuvant Actirob on the average expression level of seven NTSR markers computed over the 36 *Lolium* sp. plants studied (comparison of modalities W vs. AE, Figure 5). Considering the 17 markers for which a significant effect of the experimental modality had been detected on the expression level in this experiment (Table 3), the average expression level of 14 markers was significantly higher in the presence of iodosulfuron + mesosulfuron (comparison of modalities AEIM vs. AE, Figure 5). For these markers, the average increase in expression level ranged from 4.1-fold (*GST-tau-J*) to 31.3-fold (*GT-D*). A possible effect of the safener mefenpyr-diethyl on the expression level of the 17 markers was sought in two pairs of experimental modalities: Actirob + ethoxylated castor oil + mefenpyr-diethyl vs. Actirob + ethoxylated castor oil (comparison of modalities AEM vs. AE, Figure 5) and Actirob + ethoxylated castor oil + iodosulfuron + mesosulfuron + mefenpyr-diethyl vs. Actirob + ethoxylated castor oil + iodosulfuron + mesosulfuron (comparison of modalities AEIMM vs. AEIM, Figure 5). A significantly higher expression in the presence of mefenpyr-diethyl was observed only in the comparison of modalities AEIMM vs. AEIM for six markers (*CYP72A, CYP81B1, CYP81B2, GST-tau-J, HYDROL-B*, and *PEPTIDA*). For these markers, the average increase in expression level ranged from 2.9-fold (*CYP81B2*) to 17.8-fold (*HYDROL-B*).

In summary, a significant enhancing effect was observed with cloquintocet-mexyl or with mefenpyr-diethyl on the expression level of six NTSR marker genes (*ABC-A, ABC-B, ALDOL-B, CYP72A2, GST-tau-J*, and *HYDROL-B*), and a significant enhancing effect was observed with both safeners on the expression level of four additional markers (*CYP72A, CYP81B1, CYP81B2*, and *PEPTIDA*) (Figures 4, 5). Thus, a total of 10 of the 19 NTSR marker studied could be considered safener-responsive.

## Discussion

Safener action is generally considered highly species-specific and mostly restricted to grass crop species (Davies and Caseley, 1999; Hatzios and Burgos, 2004; Riechers et al., 2010; Kraehmer et al., 2014). Accordingly, very few studies to date had investigated safener effects on non-crop grasses, including grass weeds. This work considered *Lolium* spp. that are global troublesome grass weeds. It provides novel data on the effect of two major safeners, cloquintocet-mexyl and mefenpyr-diethyl, on the phenotypic and transcriptional response of *Lolium* sp. to ALS inhibitors that are the second most used herbicide mode of action worldwide (Kraehmer et al., 2014).

### Safener action on *Lolium* sp. sensitivity to ALS inhibitors

Previous works investigating effects of the safeners mefenpyr-diethyl and cloquintocet-mexyl on weed sensitivity to herbicides mostly addressed the grass weed *A. myosuroides* (black-grass). A benchmark study (Cummins et al., 2009) showed a reduction in the sensitivity to the ACCase inhibitor fenoxaprop of fenoxaprop-sensitive *A. myosuroides* plants in the presence of mefenpyr-diethyl. A subsequent study reported no significant and consistent effect of mefenpyr-diethyl on the sensitivity of *A. myosuroides* plants to iodosulfuron + mesosulfuron in a set of populations where NTSR to this herbicide was present or absent (Rosenhauer et al., 2016). However, considering only the experiments in this second study where a correct fit of the sensitivity data to a dose-response model was obtained (*r*^2^ ≥ 0.70) revealed a clear decrease in the average plant sensitivity to iodosulfuron + mesosulfuron in the presence of mefenpyr-diethyl in all populations.

Another study reported no effect of cloquintocet-mexyl on the sensitivity of *A. myosuroides* plants to the field rate of pyroxsulam, although cloquintocet-mexyl induced a non-significant increase in pyroxsulam degradation in the plants assayed (deBoer et al., 2011). However, this study only considered plants sensitive to the field rate of pyroxsulam and did not include a dose-response assay that would have allowed observing or excluding an effect of cloquintocet-mexyl on plant sensitivity to pyroxsulam.

Herein, using a single herbicide dose (the French recommended field rate), we observed a clear decrease in the sensitivity of *Lolium* sp. plants to pyroxsulam and to iodosulfuron + mesosulfuron caused by cloquintocet-mexyl and by mefenpyr-diethyl, respectively (Figure 2). This effect was confirmed on individual plants sensitive or moderately resistant to the respective field rates of these herbicides (Figure 3, Table 2). The time-consuming plant cloning procedure only enabled to study one single herbicide rate. It was thus not possible to determine whether cloquintocet-mexyl or mefenpyr-diethyl caused a change in sensitivity in plants showing no phenotype shift at the herbicide field rate in the presence of safener, be they sensitive or resistant (plants in classes A or D, Table 2). The possible intrinsic responsiveness of *Lolium* sp. to safener action can thus not be excluded. Our results demonstrate that the respective field rates of cloquintocet-mexyl and of mefenpyr-diethyl could reduce, in some cases substantially, the sensitivity of individual *Lolium* sp. plants to the associated herbicides, thereby extending the results obtained with ACCase inhibitors and confirming the trends observed for ALS inhibitors in *A. myosuroides*.

### Safener action on the transcription level of NTSR marker genes

In a previous work (Duhoux et al., 2017), we showed that the 19 NTSR markers studied herein were constitutively more highly expressed in plants with NTSR than in sensitive plants, and that application of the French field rate of the commercial formulation of iodosulfuron + mesosulfuron associated with mefenpyr-diethyl also used herein caused a substantial increase in NTSR marker expression 48 h after application. Herein, we observed that expression of most NTSR markers was strongly increased by the herbicides pyroxsulam or iodosulfuron + mesosulfuron in the absence of their associated safeners (Figures 4, 5). Our results thus confirm that NTSR markers are herbicide-responsive, thereby confirming their relevance.

Data concerning genes which expression level is modified by safeners is scarce in weeds. Basically, only conjugating enzymes involved in the phase II of herbicide metabolism had previously been reported as safener-responsive in weeds. Mefenpyr-diethyl and cloquintocet-mexyl caused an increased expression of genes encoding glutathione-*S*-transferases in *A. myosuroides* (Cummins et al., 2009) and in the weedy wheat ancestor *Aegilops tauschii* (Zhang et al., 2007), respectively. Other safeners not investigated in our work had also been reported to cause an increased expression of genes encoding glutathione-*S*-transferases in *A. myosuroides* (Cummins et al., 1999; Reade et al., 2004), in the non-crop grass *Festuca arundinacea* (Del Buono et al., 2007) and in the grass *Lolium multiflorum* that is both a weed and a crop (Del Buono and Ioli, 2011). In *L. multiflorum* and *A. myosuroides*, glutathione-*S*-transferase induction by safeners was associated to a reduction in herbicide sensitivity (Cummins et al., 1999, 2009; Reade et al., 2004; Del Buono and Ioli, 2011). The possible action of two safeners, including cloquintocet-mexyl, on the activity of O-glucosyl-transferases (phase II) had been investigated in *A. myosuroides*, but no effect could be observed (Brazier et al., 2002). Herein, we observed that 10 of the 19 NTSR marker genes studied could be considered safener-responsive (i.e., their expression was increased in the presence of at least one of the two safeners studied) (Figures 4, 5). These markers were predicted to code for enzymes or proteins potentially involved in the four phases of herbicide metabolism: four cytochromes P450 and one hydrolase (phase I), one glutathione-*S*-transferase (phase II), two ABC transporters (phase III) and one peptidase (phase IV). Our results thus suggest that both safeners triggered a coordinated induction of some herbicide detoxification pathways, probably through transcriptional activation of genes involved in the four phases of herbicide metabolism in *Lolium* sp. This is consistent with previous results obtained in model or crop plants (Wolf et al., 1996; Hatzios and Burgos, 2004; DeRidder and Goldsbrough, 2006; Zhang et al., 2007; Skipsey et al., 2011).

Four of the 10 safener-responsive NTSR markers showed a significantly higher expression level in the presence of both safeners. This suggested that, although chemically unrelated, cloquintocet-mexyl and mefenpyr-diethyl stimulated identical or strongly overlapping secondary metabolism pathways in *Lolium* sp.. These results are consistent with a previous work demonstrating that cloquintocet-mexyl and mefenpyr-diethyl caused accumulation of identical sets of glutathione-*S*-tranferases in wheat (Taylor et al., 2013). They also indicate that mechanisms of safener action are highly similar in weeds and in crop plants.

An enhancing effect of safeners on safener-responsive NTSR markers was expected when applying the safener alone and together with its associated herbicide. However, this was only observed for one marker with cloquintocet-mexyl (*ABC-B*, Figure 4) out of the eight NTSR markers with an expression significantly increased by cloquintocet-mexyl and the six markers with an expression significantly increased by mefenpyr-diethyl. A significant enhancing effect of cloquintocet-mexyl was observed for five NTSR markers when the safener was applied alone, but not when it was associated to pyroxsulam (Figure 4). This could be due to pyroxsulam alone having enhanced the expression of these markers to a level where it cannot be further increased by cloquintocet-mexyl. A limit to the expression level of safener-responsive genes had been previously observed in *A. myosuroides*: the effect of mefenpyr-diethyl on the expression level of glutathione-*S*-transferases modulating sensitivity to one ACCase inhibitor was proportionally lesser on plants with constitutively high expression of these genes than on plants with weak or no expression (Cummins et al., 2009). The two last NTSR markers with an expression significantly increased by cloquintocet-mexyl and the six markers with an expression significantly increased by mefenpyr-diethyl only showed increased expression when the safener was associated to the corresponding herbicide (Figures 4, 5). This could be because safeners alone have little enhancing effect on the expression of these markers, but can amplify their induction by the associated herbicide. In this hypothesis, there would be two types of safener-responsive herbicide degrading pathways: pathways that could be activated by safeners and/or herbicides, and herbicide-activated pathways that could be further exacerbated by safeners. Another possibility would be a transient exacerbating effect of the safener alone that would not persist until 24 h after treatment, when gene expression level was measured in our experiments. Dedicated experiments including measurement of NTSR marker gene expression over time are requested to check these hypotheses.

In summary, our results indicate that both cloquintocet-mexyl and mefenpyr-diethyl likely had an enhancing action on some herbicide-responsive secondary metabolism pathways involved in NTSR. This is in agreement with results obtained in crop or model species showing that plant secondary metabolism pathways involved in herbicide response are present among the pathways activated by safeners (Davies and Caseley, 1999; Hatzios and Burgos, 2004; Riechers et al., 2010). We conclude that safeners most likely have an action on *Lolium* sp. similar to that observed on crop species, i.e., they enhance herbicide degradation by coordinately activating herbicide metabolism pathways, thereby increasing *Lolium* sp. detoxifying response to herbicides without increasing herbicide toxicity.

### A role for safeners in the evolution of NTSR?

While safeners have been predominantly used in the field to increase the selectivity of one particular herbicide toward crop plant(s), their protective action extends to multiple classes of herbicide chemistries or modes of action (Riechers et al., 2010; Kraehmer et al., 2014; Rosinger, 2014). Cloquintocet-mexyl and mefenpyr-diethyl have first been associated to herbicides inhibiting ACCase used to control *Lolium* sp. in cereals (Riechers et al., 2010; Kraehmer et al., 2014; Rosinger, 2014). These associations have been the major herbicides used to control grass weeds in cereals in France in the 1990s-early 2000s. Their intensive use selected for resistance essentially due to NTSR in numerous *Lolium* sp. populations (Kaundun, 2014), including those analyzed here (about 10 and 90% of the plants in populations RG07-044 and RG07-046, respectively, were resistant to the ACCase inhibitors clodinafop and/or pinoxaden because of NTSR). Evolution of resistance was one major reason why ACCase inhibitors were supplanted by the ALS inhibitors iodosulfuron + mesosulfuron and pyroxsulam in French fields in the late 2000s. However, like ACCase inhibitors, iodosulfuron + mesosulfuron and pyroxsulam have been used in association with mefenpyr-diethyl and cloquintocet-mexyl, respectively. In commercial herbicide formulations, each safener was used with ALS inhibitors at a field rate about 1.2-fold higher than with ACCase inhibitors (22.5 g/ha mefenpyr-diethyl associated to iodosulfuron + mesosulfuron vs. 18.7 g/ha associated with the ACCase inhibitor fenoxaprop, and 18.75 g/ha cloquintocet-mexyl associated to pyroxsulam vs. 15 g/ha in association with the ACCase inhibitors clodinafop or pinoxaden). Thus, *Lolium* sp. populations subjected to the selective pressure of ACCase inhibitors associated with cloquintocet-mexyl or mefenpyr-diethyl were subsequently sprayed with ALS inhibitors associated with a higher field rate of the same safeners. Some of the mechanisms involved in NTSR to ACCase inhibitors are also involved in NTSR to ALS inhibitors (Beckie and Tardif, 2012). Furthermore, previous studies had shown that safener effect can be dose-dependent (Rosinger, 2014; Rosenhauer et al., 2016).

To enable weed plant survival, NTSR mechanisms must allow rapid and efficient herbicide neutralization (Yuan et al., 2007; Délye, 2013). Herbicide selective pressure in the field is thus expected to favor plants showing high constitutive expression of herbicide-neutralizing pathways (i.e., constitutive NTSR) and/or rapid and strong activation of such pathways in the presence of herbicides (i.e., herbicide-induced NTSR). NTSR had been proposed to evolve in weed populations by a gradual increase in the expression of NTSR pathways over weed generations under the herbicide selective pressure (reviewed in Délye et al., 2013; see also Duhoux et al., 2017). In our experiments, the effect of safeners on individual plant sensitivity was identical between the two clones analyzed per individual plant (illustrated in Figure 3) but varied among plants. This clearly indicated that genetic variation for safener response is present in *Lolium* sp. This finding, the literature data and the history of safener use in the field concur to suggest another possible pathway for weed evolution toward NTSR could be by selection for increased responsiveness to safener action. This selection could occur together with selection for exacerbated herbicide metabolism, and possibly facilitate it. This hypothesis needs to be addressed, for instance by comparing the outcome of selection using one herbicide alone or in association with one safener.

## Conclusion

Our results add to the growing body of evidence contradicting the widespread assumption that safener action, or at least safener action at one given dose, would be crop-specific (Riechers et al., 2010; Kraehmer et al., 2014). Here, using two broadly used safeners at their respective recommended field rates, we demonstrated a reduction in the sensitivity of *Lolium* sp. plants to ALS inhibiting herbicides. Clearly, safeners are a two-edged sword in terms of weed control. On one hand, they enable control of weeds in botanically closely related crops using a diversity of herbicide modes of action. On the other, they might facilitate the evolution of NTSR in these weeds. Another issue with safeners is that they are compounds with substantial biological activity, but which environmental fate and possible toxicity are poorly known (Sivey et al., 2015). Technical solutions, such as delivering the safener exclusively to the crop, which could be done by technologies such as seed coating or targeted spraying (Riechers et al., 2010; Kraehmer et al., 2014) should be considered to mitigate the potential resistance and environmental risks induced by associating safeners to herbicides.

Our results indicate that safeners associated to ALS inhibitors reduce the sensitivity of *Lolium* sp. plants to their associated herbicides by exacerbating already existing NTSR pathways. From this point of view, safeners show an intriguing potential to address the mechanisms governing NTSR in weeds, and particularly to try and identify determinants of NTSR regulation that remain unknown to date. Safeners could also be used pro-actively to try and identify metabolic pathways involved in NTSR to future herbicides before they are commercially released, which would help in implementing anti-resistance strategies or developing “resistance-breaking” technologies that would hamper resistance evolution in the field. Unravelling NTSR mechanisms in weeds by using safeners is an option that remains largely unexplored. The basic patents of many safeners, including cloquintocet-mexyl and mefenpyr-diethyl, having now expired (Riechers et al., 2010) should facilitate future safener-based NTSR research.

## Author contributions

AD and CD designed this study. AD and DD produced and treated the plant material in the greenhouse and carried out the plant phenotype rating. AD, DD, and FP performed the molecular biology analyses. AD and CD analyzed the data and wrote the manuscript. CD supervised the project. All authors read and approved the manuscript.

### Conflict of interest statement

The authors declare that the research was conducted in the absence of any commercial or financial relationships that could be construed as a potential conflict of interest.
